# Shell-type acoustic metasurface and arc-shape carpet cloak

**DOI:** 10.1038/s41598-019-44619-z

**Published:** 2019-05-30

**Authors:** Fuyin Ma, Yicai Xu, Jiu Hui Wu

**Affiliations:** School of Mechanical Engineering & State Key Laboratory for Strength and Vibration of Mechanical Structure, Xi’an Jiaotong University, Xi’an, 71009 China

**Keywords:** Mechanical engineering, Acoustics

## Abstract

We systematically propose a thin shell-type acoustic metasurface, which could be used to design a carpet cloak that closely covers an arc-shaped object, therefore providing the necessary support for hiding an object with any arbitrary shape. To facilitate the experimental measurement, however, the work here starts with some rotary spherical shell-type and ellipsoidal shell-type cell structures. The measured and calculated sound transmission loss (STL) results of these structures suggest that the sound insulation performances of the shell-type structure are quite different from those of the plate-type structure, indicating a possible break in the shape of the classical sound insulation curve. Considering also that cylindrical shell structures are more widely used in practice than the rotary shell structures, a number of two-dimensional bilayer cylindrical and elliptic cylindrical shell structures were, therefore, designed in this assay. Due to the asymmetry of the structure, the shell-type cells could exhibit bianisotropic sound absorption, reflection and effective parameters. Furthermore, the stiffness of the thin shell structure changed nonlinearly with the changing of the radius of curvature, with a wing shape tendency. In addition, a bilayer cylindrical shell-type acoustic metasurface and an arc-shaped carpet acoustic cloak were successively designed, wherein the phased compensation of differently shaped cell structures could be adjusted by means of a new engineering iso-phase design method. This work could provide the necessary guidance to extend existing results in the field of membrane- and plate-type acoustic metamaterials for shell-type structures, and the realization of the arc-shaped cloak could provide support for the design of a carpet acoustical cloak for use with arbitrary shapes.

## Introduction

Acoustic metamaterials are man-made composite media structured on a scale much smaller than a wavelength. Through ingenious micro-structure design, acoustic metamaterials can exhibit acoustic bandgap^1^, negative effective parameters^2–5^, total reflection^2^, anomalous reflection/refraction^6–9^, perfect absorption^10–12^, focus^13,14^, abnormal Doppler effect^15^, non-reciprocal acoustic transmission^16,17^ and many other peculiar physical phenomena. As a result, acoustic metamaterials have been utilized in numerous promising applications, including vibration and noise suppression^18–20^, imaging^21,22^, superlens^23,24^, waveguide^25,26^, cloak^27–32^ and other aspects of the acoustics field. As a kind of artificial composite structure, it has the potential to realize expected functions through microstructure designs^33–38^. In these special functional devices, lightweight membrane and plate-type structures have attracted the attention of engineers for low-frequency sound attenuations and other applications due to their ultra-thinness and excellent sound wave manipulation abilities in low frequency ranges^2,5,10,18–20^. In engineering practice, however, the shell structures are more widely used than the plate and membrane structures. In fact, the vibration characteristics of membrane and plate are similar, while those of shell are markedly different, and further research into shell structures is urgently needed in order to realize the complex shape of this acoustic metamaterial structure design.

In a quest to further reduce the size of structures in recent years, metasurfaces have been developed from existing metamaterials to manipulate acoustic waves in a small scale space and achieve arbitrary control of the amplitudes and phases of wavefronts^39^. Acoustic metasurface can be used to achieve full control of wavefronts, thereby further realizing developments such as an acoustical planar focusing superlens^13,14^, wavefront self-collimation^6,8^, redirections^6–9^ and cloaks^27–32^. In general, the building units of acoustic metasurfaces include resonant membranes/plates, Helmholtz resonators, or space-coiling units^40^. Among the numerous types of acoustic cloaks, the thickness of a carpet cloak is sufficiently small and it has a good application prospect. However, the carpet cloak’s commonly used cells belong to planar structures, so they can only approximately realize a design for objects with arbitrary shapes through the approximation of an arc curve by straight line when the curvature of the cell coverage area is small^41^. When the curvature of an object is large, it is impossible to obtain a carpet-type cloak that completely matches the object’s surface. For this reason, in this paper we develop our previously proposed bilayer plate-type acoustic metasurface into a shell-type structural system, and propose a shell-type acoustic metasurface to realize an arc-shape acoustic cloak. Each innovative new shell structure can be designed according to the surface curve shape of the object, so it may realize a carpet cloak that completely matches the surface, and provides the possibility of designing carpet cloaks with arbitrary shapes.

## Breaking the Cutoff Frequency in STL Curve by Shell-type Structure

Plates and shells, two basic types of thin-walled structures, are both commonly used in industrial equipment, however, their mechanical behaviors and vibration characteristics are markedly different. Assuming the lateral vibration displacement of thin plate is *w*, then the bending vibration equation of homogeneous plate is:

1 $$D(\frac{{\partial }^{4}w}{\partial {x}^{4}}+2\frac{{\partial }^{4}w}{\partial {x}^{2}\partial {y}^{2}}+\frac{{\partial }^{4}w}{\partial {y}^{4}})+{m}_{s}(\frac{{\partial }^{2}w}{\partial {t}^{2}})=0$$

where *m*_*s*_, *D*, *E*, *μ* and *h* are the surface density, bending stiffness, Young’s modulus, Poisson’s ratio and thickness of thin plate respectively, and $D=\frac{E{h}^{3}}{12(1-{\mu }^{2})}$.

And the vibration equation of rotary shell is^42^:

2 $$\{\begin{array}{rcl}\rho h\frac{{\partial }^{2}u}{\partial {t}^{2}} & = & K(\frac{{\partial }^{2}u}{\partial {x}^{2}}+\frac{1-\mu }{2{r}^{2}}\frac{{\partial }^{2}u}{\partial {\theta }^{2}}+\frac{1+\mu }{2r}\frac{{\partial }^{2}v}{\partial x\partial \theta }+\frac{\mu }{r}\frac{\partial w}{\partial x})\\ \rho h\frac{{\partial }^{2}v}{\partial {t}^{2}} & = & K(\frac{1+\mu }{2r}\frac{{\partial }^{2}u}{\partial x\partial \theta }+\frac{1-\mu }{2}\frac{{\partial }^{2}v}{\partial {x}^{2}}+\frac{1}{{r}^{2}}\frac{{\partial }^{2}v}{\partial {\theta }^{2}}+\frac{1}{{r}^{2}}\frac{\partial w}{\partial \theta })+\frac{D}{{r}^{2}}(\frac{1}{{r}^{2}}\frac{{\partial }^{2}v}{\partial {\theta }^{2}}+\frac{(1-\mu )}{2}\frac{{\partial }^{2}v}{\partial {x}^{2}}-\frac{{\partial }^{3}w}{\partial {x}^{2}\partial \theta }-\frac{1}{{r}^{2}}\frac{{\partial }^{3}w}{\partial {\theta }^{3}})\\ \rho h\frac{{\partial }^{2}w}{\partial {t}^{2}} & = & -K(\frac{\mu }{r}\frac{\partial u}{\partial x}+\frac{1}{{r}^{2}}\frac{\partial v}{\partial \theta }+\frac{1}{{r}^{2}}w)+D(\frac{1}{{r}^{4}}\frac{{\partial }^{3}v}{\partial {\theta }^{3}}+\frac{1}{{r}^{2}}\frac{{\partial }^{3}v}{\partial {x}^{2}\partial \theta }-\frac{{\partial }^{4}w}{\partial {x}^{4}}-\frac{1}{{r}^{4}}\frac{{\partial }^{4}w}{\partial {\theta }^{4}}-\frac{2}{{r}^{2}}\frac{{\partial }^{4}w}{\partial {x}^{2}\partial {\theta }^{2}})\end{array}$$

where *ρ*, *h*, *μ*, *K*, *E* and *D* are the mass density, thickness of shell, Poisson’s ratio, membrane stiffness, Young’s modulus, and bending stiffness, and $K=\frac{Eh}{1-{\mu }^{2}},\,D=\frac{E{h}^{3}}{12(1-{\mu }^{2})}$.

It can be seen from the Eqs 1, 2 that the vibration equation of the shell is much more complicated than that of the thin plate. Moreover, the stiffness of shell structures includes both membrane stiffness and bending stiffness, while only bending stiffness is considered in plates. These different vibration characteristics are originated from the different geometric symmetries between plate and shell, and would inevitably lead to different acoustic properties in the metamaterial structures composited by shells compared to those composited by plates.

**Figure 1:**
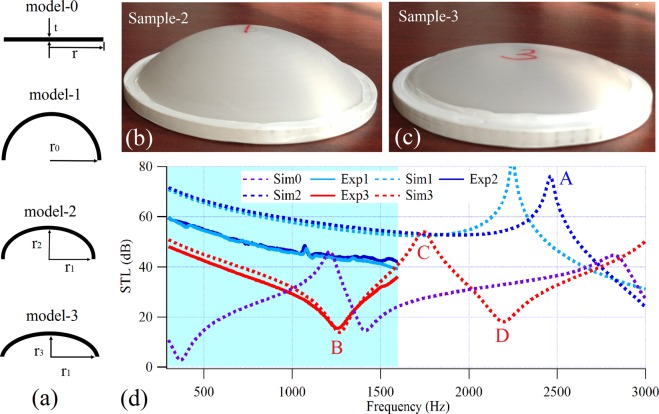
Models, experimental samples and measured results. (**a**) The structure diagram of models (front view); the photograph of (**b**) sample-2 and (**c**) sample-3; (**d**) the simulated and measured STL curves of the model-0 (Sim0), model-1 (Sim1 and Exp-1 for simulated and experimentally measured results, respectively), model-2 (Sim2 and Exp-2) and model-3 (Sim3 and Exp-3), wherein the shaded region denotes the measurement frequency range.

Two types of thin-shell structures are most commonly used, namely rotary shell and cylindrical shell, with the latter more widely applied. Cylindrical shell structures are inconvenient for sound transmission measurements, therefore, we first performed our experimental verification using rotary shell structures. For comparison, two models (model-0 and model-1), including a thin plate and a hemispherical rotary shell, were designed respectively. Model-0 was a circular plate with thickness *t* = 1 mm and radius *r* = 50 mm, while model-1 was a hemispherical rotary shell with a radius of *r*_0_ = 45 mm. In addition, due to the spherical rotary shell’s greater level of stiffness, which made it difficult to obtain clear resonance and anti-resonance features in the measurement frequency range, two ellipsoidal rotary shell structures with different short-axis radii (model-2 and model-3) were also established. In these, the identical long-axis radius of each was *r*_1_ = 45 mm, the short-axis radius of model-2 was *r*_2_ = 30 mm, and that of model-3 was *r*_3_ = 10 mm. The cross-sectional schematic diagrams (front view) of the above four structures are shown in Fig. 1a. The identical thickness of both plate and shells was *t* = 1 mm. Three samples, corresponding to model-1, model-2 and model-3, were fabricated using 3D printing technology. The samples corresponding to model-2 and model-3 are shown in Fig. 1b,c, respectively. It should be noted that, to facilitate the measurement, a ring with an inner radius of 45 mm, an outer radius of 50 mm and a thickness of 5 mm was added to the outer side of the shells. The STLs of samples were measured by means of a Bruel & Kjaer Type-4206T impedance tube system and compared with the calculated results in order to demonstrate the transmission properties of the plate and shell structures, as seen in Fig. 1d. Using the acoustic-solid coupling module of a commercial finite element software, Comsol Multiphysics 4.3a, the corresponding models were then solved by simplification into two-dimensional symmetric models. In order to fully capture the resonance and anti-resonance features, the calculation frequency range was set to 300–3000 Hz, and the measurement range was limited by equipment to 300–1600 Hz. A photosensitive resin material was used to fabricate the samples, for which the Young’s modulus, Poisson’s ratio and mass density were (2.2 + 0.11i) GPa, 0.375 and 1000 kg/m^3^, respectively. The imaginary part of the Young’s modulus represents the viscoelastic damping loss of the materials (damping ratio is 5%).

The STL curves in Fig. 1d demonstrate the excellent sound attenuation abilities of these shell-type units, especially at the maximum STL value of Sim1, which reaches up to approximately 80 dB. Throughout the entire measurement range, Exp3 and Sim3 were found to agree well with each other. Moreover, although the amplitudes of both Exp1 and Exp2 were approximately 12 dB lower than those of Sim1 and Sim2, the trends of their curves are similar, and both the measurement and simulation results exhibit the strong sound insulation performance with attenuations higher than 99%. Obviously, the calculated sound insulation values are higher than those of measured. The main reason is that the sample boundary was perfectly fixed in the calculations, while in the experiments the sample was installed in tube under an interference fit through some softer sealing materials on sample boundary. Therefore, in the experiment, the sample boundary is equivalent to an elastic support condition. This causes the localized stiffness in the experiments is lower than that in the simulations. In the stiffness control region of STL curve, the amplitude is mainly determined by the localized stiffness provided by the boundary condition, so the amplitudes of the measurement results, including Exp1, Exp2 and Exp3 are lower than the corresponding calculation results. Overall, the results suggest that, because the stiffness of a shell is significantly higher than that of a plate, shell-type structures will provide a superior sound insulation performance than plate-type structures with the same thickness. In particular, when the curvature of the shell is large, the sound insulation performance becomes excellent in all low-frequency and middle-frequency ranges, with no obvious STL dip. This means that replacing a plate-type structure with a shell-type structure could significantly improve low-frequency sound insulation performance. Additionally, as the cutoff frequency feature between the stiffness control region and the damping control region of the sound insulation curve can be breaking, the STL dip at cutoff frequency was eliminated. In Exp1 and Exp2, the average STL of each in the measurement range reached approximately 50 dB, while for Sim1 and Sim2, it reached 70 dB in the frequency range below 3000 Hz. Both the experiment and calculation results, therefore, exhibit unparalleled excellence in broadband sound insulation abilities.

As the radius of curvature increases, the dimensional characteristics difference between the shell and the plate decreases, and the vibration characteristics of the shells become closer to those of the plates. By comparing Sim0 and Sim3, it can be seen that as the radius of curvature decreases, the stiffness of the shell structure increases, resulting in an increase in the frequencies of both the first dip and peak. The first dip (peak) frequency of Sim0 is 368 Hz (1212 Hz), while that of Sim3 is 1268 Hz (1748 Hz). In addition, the STL values at the dip and peak of the shell structure are also much higher than those of the plate structure. The first dip (peak) value of Sim0 is 2.8 dB (46.1 dB), and that of Sim3 is 13.9 dB (54 dB). In a comparative analysis of Sim1, Sim2 and Sim3, it is evident that the stiffness of the shell structure does not change linearly with the curvature. The first anti-resonance peak of Sim2 is higher than that of Sim1, wherein the former is 2460 Hz, while the latter is 2244 Hz. The above measurement and calculation results show that, on the one hand, the sound insulation characteristics of the shell structure are distinctly different from those of the plate structure. As the radius of curvature decreases, the boundary between the stiffness control region and damping control region of the traditional sound insulation curve disappears, and the STL dip at the cutoff frequency is eliminated. On the other hand, the stiffness of the shell structure is larger than that of the plate structure, but the stiffness does not change linearly with the radius of curvature. Most importantly, although the structures are of the same thickness, the sound insulation performance of the shell structure is significantly better than that of the plate structure and, by reducing the radius of curvature, can be achieved across the entire low-frequency and middle-frequency ranges. It should also be noted that the surface density of the shell is not much larger than that of the plate, and that it is a lightweight structure.

**Figure 2:**
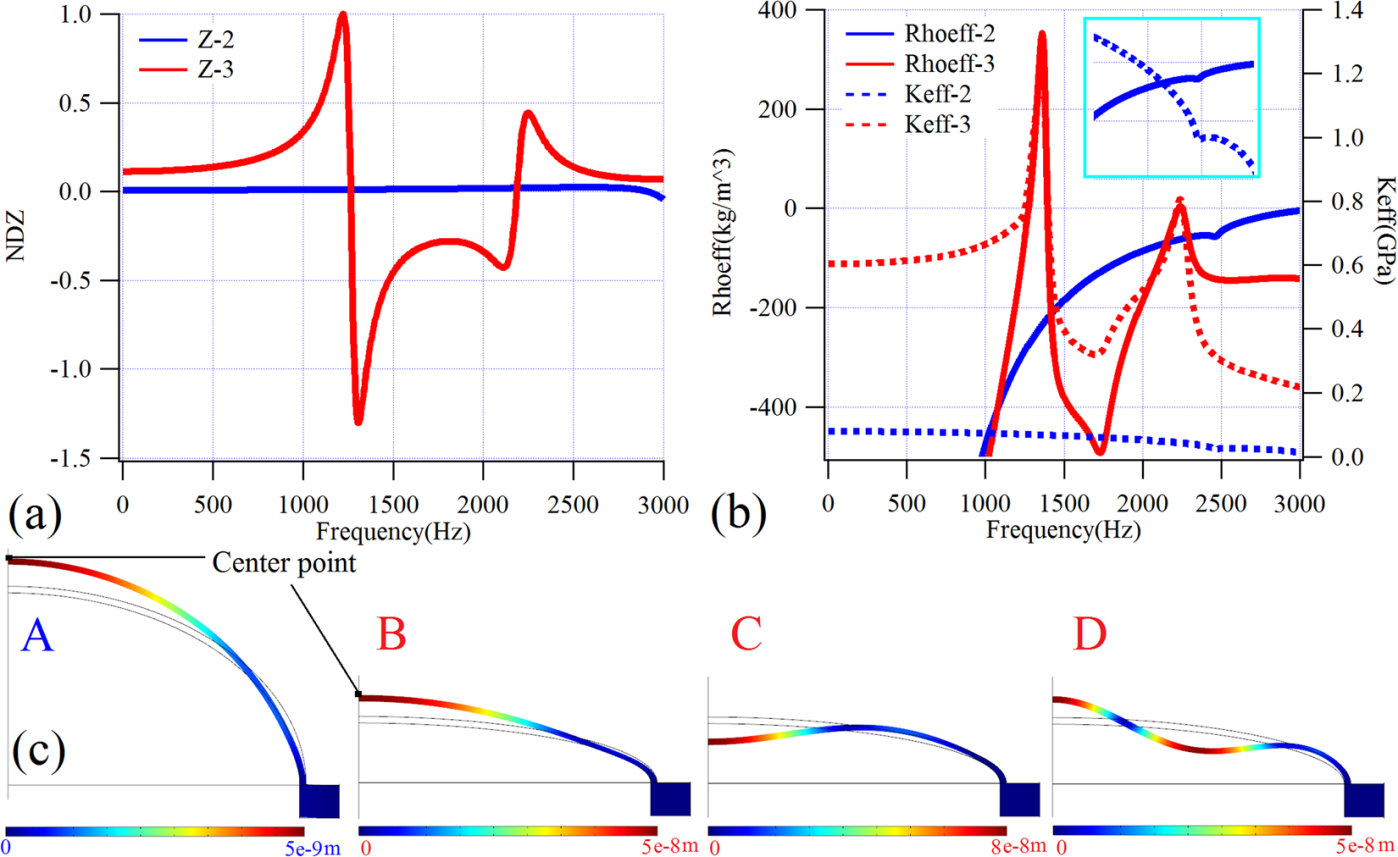
Calculated displacement results, effective parameters and displacement profiles. (**a**) The normalized displacements in Z direction (NDZ) at the center point of model-2 and model-3; (**b**) the effective mass densities and the effective moduli of the models 2 and 3. (**c**) The displacement profiles at the points marked in Fig. 1d.

In order to reveal the physical mechanism by which the cutoff frequency in the sound insulation curve is broken by the thin-shell structure, we selected model-2 and model-3 with which to calculate the normalization displacement of the thin-shell in the Z-direction (using the largest positive value Z3 normalizes all data) at the center point (Fig. 2c). The results are shown in Fig. 2a, in which it can be seen that, at the first sound STL dip (cutoff frequency) of model-3, the displacement of the center point drops dramatically from a maximum positive value to a maximum negative value. In considering also the displacement profile (point-B, 1268 Hz) in Fig. 2c, it is evident that, at this frequency, the displacement of the first order resonance reaches a maximum value accompanying with a mode transition. Similarly, at the second STL dip, the displacement produces a sharp rise from a maximum negative value to a maximum positive value. By considering also the displacement profile (point-D, 2200 Hz) in Fig. 2c, it can be seen that, at that frequency, the displacement of the second order resonance reaches a maximum value accompanying with a mode transition. In addition, from the displacement profile at point-C in Fig. 2c, it can be seen that, similar to the plate- and membrane-type structures, at the first STL peak, the shell is anti-resonant and reaches maximum displacement, thus initiating a mode transition. However, as can be seen from Fig. 2a, the displacement at the center point of model-2 changes uniformly across the whole calculation frequency range and no large shift occurs. This trend suggests that the mode transition point should occur at a higher frequency, and the displacement of the center point indicates that the cutoff frequency of the model-2 has not yet arrived in the calculation frequency range. Furthermore, in combination with the displacement field (point-A) in Fig. 2c, it can be seen that, at the STL peak, the vibration mode of the structure is still resonant and no mode transition occurs. This indicates that the STL peak at point-A in Fig. 1d is not produced by the anti-resonance of the structure. In order to reveal the mechanism of this STL peak, the effective mass density and bulk modulus of the two models were solved by employing the retrieval method^43^, as plotted in Fig. 2b. It can be seen from the figure that for model-3 the effective mass densities are zero at the two STL dips, and both the effective mass density and bulk modulus reach a dip at the STL peak. For model-2, the effective mass density remains negative in the calculated frequency band, while the effective bulk modulus remains positive, and no sign change occurs. However, at the STL peak of point-A, both the effective mass density and the effective bulk modulus have an inflection point. That is, the STL peak is caused by discontinuity effective parameters, which is completely different from the conventional STL peak generated by the anti-resonance mode. These results imply that the change in the shape of the sound insulation curve in Fig. 1c is produced by the creation of a new STL peak in the stiffness control region, rather than by eliminating the cutoff frequency. It should be noted that the STL peak herein is equivalent to a further superimposing effect in the original stiffness control region of high STL values, thereby obtaining an excellent broadband low-frequency and middle-frequency sound insulation performance.

## Bianisotropic Acoustical Properties of Shell-type Structure

**Figure 3:**
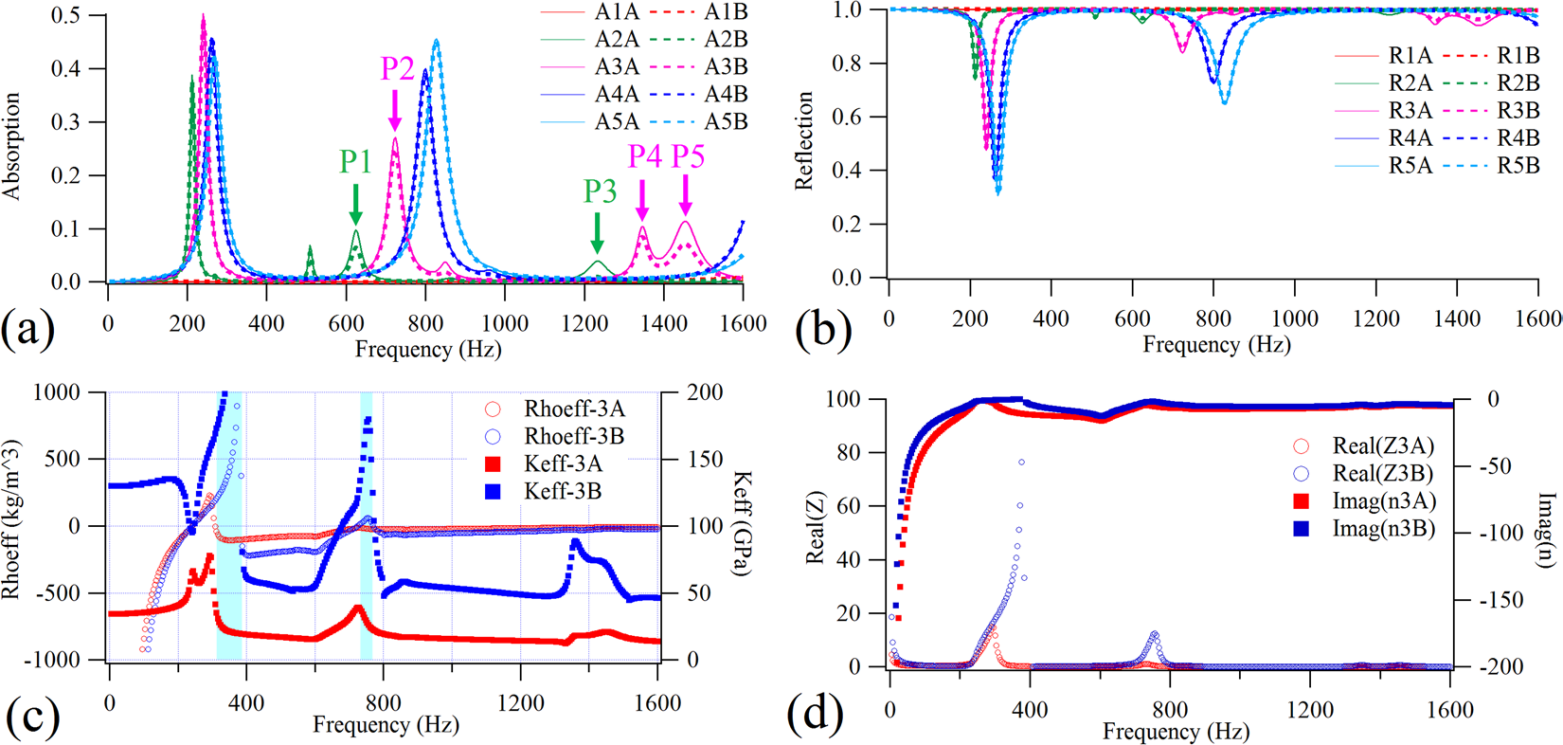
Bianisotropic sound absorptions, reflections and effective parameters. The calculated sound (**a**) absorption coefficients and (**b**) reflection coefficients of structures with different radius of curvature and opposite sound incident directions. (**c**) The effective mass densities and bulk modulus of the 3# structure with opposite sound incident directions. (**d**) The real part of equivalent impedance (Z) and imaginary part refractive index (n) of the 3# structure with opposite incident directions.

In engineering practice and in daily life, cylindrical shells are more widely used than rotary shells. The fuselages and bodies of airplanes, automobiles and trains, for example, are composed mainly of cylindrical shells. In order to provide a wider range of guidance for engineering applications, in this section, cylindrical shell-type structures are considered. The shell is clearly an asymmetrical structure and, according to previous studies, must exhibit bianisotropic acoustic properties^38^. Here, bianisotropic behavior means that, due to its asymmetry, the structure exhibits different acoustic properties when sound waves irradiate from its different sides. According to the principle of reciprocity, for a passive system, the transmission is changeless regardless of the side from which the incident sound wave irradiates. This direction-dependent acoustic property is known as bianisotropic, rather than asymmetric, behavior^44^. Structures with bianisotropic acoustic characteristics could create challenges for the solution of effective parameters and, therefore, bianisotropic behavior has become a hot research topic in the field of acoustic metamaterials. In this study, in order to reveal the bianisotropic acoustical properties of shell-type structures, a type of bilayer cylindrical shell-type acoustic metamaterial with a different short-axis radius was designed. The absorption and reflection coefficients of the structures were calculated and are shown in Fig. 3a,b, respectively. The radius of the long-axis was kept at 50 mm and five sets of short-axis radius parameters were selected, wherein the short-axis radius of the 1# cell was 50 mm, 40 mm for 2#, 30 mm for 3#, 20 mm for 4#, and 10 mm for 5#. The sound absorption and reflection coefficients of the structures were calculated when the incident sound waves perpendicularly irradiate from the concave side and the convex side, respectively. Herein the incidence sound wave irradiating from the concave side of the shell was denoted as A, while that from the convex side was denoted as B. In Fig. 3a,b, A1A (A1B) represents the sound absorption coefficient of the 1# cell for the incident sound wave irradiating from the A (B) side, and R1A (R1B) represents the reflection coefficient of the 1# cell for the incident sound wave irradiating from the A (B) side. The other structures are analogized in turn. A type of aluminum shell with a 0.2 mm-thickness was used here, for which the Young’s modulus, Poisson’s ratio and mass density were (70 + 3.5i) GPa, 0.28 and 2800 kg/m^3^, respectively. The two aluminum shells were separated by a 0.2 mm air layer. As can be seen from the figure, regardless of which side the sound wave irradiates from, the absorption coefficients of the hemispherical shell-type cell (1#) in the calculated frequency range are close to 0, and the reflection coefficients are close to 1, showing a broadband total reflection feature. As the radius of the short-axis decreases, the bilayer shell-type structure exhibits a certain sound absorption capacity, which can reach up to 0.5. As the figure clearly shows, the sound absorption coefficients of 2# cell and 3# cell have an obvious orientation dependence characteristic, especially at the sound absorption peaks corresponding to P1, P2, P3, P4 and P5. While the other cells have almost no orientation dependence characteristic, the features of the reflection are similar to those of absorption. This means that the bianisotropic sound absorption and reflection characteristics exist only within a specific short-axis radius range. The sound absorption results suggest that the absorption amplitude and frequency position of the first absorption peak do not change monotonously with the short-axis radius. Instead, the frequency position of the second peak will increase as the short-axis radius decreases, and the absorption amplitude will also increase. The above results indicate that, on the one hand, the sound absorption and reflection coefficients of the shell-type structure will change with the radius of the shell curvature; on the other hand, the absorption and reflection coefficients of the multilayer shell-type structure are clearly bianisotropic within a certain radius of curvature range.

In previous work, we studied an asymmetrical bilayer plate-type structure, which exhibits bianisotropic sound absorption, reflection and an effective parameters feature^38^. It is known from the retrieval method for solving the effective parameters that the effective mass density and bulk modulus are determined by the transmission and reflection coefficients^43^. According to the reciprocity principle, the transmission coefficient is changeless regardless of the side from which the incident sound wave irradiates, which means that the bianisotropic reflection coefficient can lead to bianisotropic effective parameters. Taking the 3# cell with obvious bianisotropic characteristics as an example, we solved the effective mass density and bulk modulus of the bilayer shell-type structure under different sound incident directions using the retrieval method. The results, shown in Fig. 3c, suggest that Rhoeff-3A and Rhoeff-3B are basically coincident with each other in the frequency range below 300 Hz, while in the two frequency bands (shaded areas) near the first two absorption peaks their symbols are opposite, and in the entire frequency range above 300 Hz, the differences between the two groups are large, showing a clear bianisotropic effective mass density feature. Moreover, both the Keff-3A and Keff-3B have positive values in the entire calculated frequency range, although the differences between them are obvious, showing a clear bianisotropic effective bulk modulus feature. It should be noted that, when using the retrieval method in the literature to solve the effective parameters, it was necessary to satisfy the conditions that the real part of the effective impedance should remain positive and the imaginary part of the refractive index should remain negative. For this purpose, we calculated the real part of the effective impedance and the imaginary part of the refractive index of the 3# cell under opposite sound incident directions, as shown in Fig. 3d. It was found that the above two conditions are always satisfied in the calculation frequency range, which illustrates the validity of the calculated effective parameter results. In theory, the bianisotropic behavior originates from the scattering properties of sound waves. In the case of the thin shell, the incident sound waves irradiating from both the convex side and the concave side will encounter different structure surfaces, so the scattering sound fields will be different. This will result in different reflection coefficients when the sound waves are incident from different sides, and the sound absorption coefficient will also be different, followed by different effective parameters. This confirms that the source of the bianisotropic acoustic properties is the asymmetric geometric of the structure.

**Figure 4:**
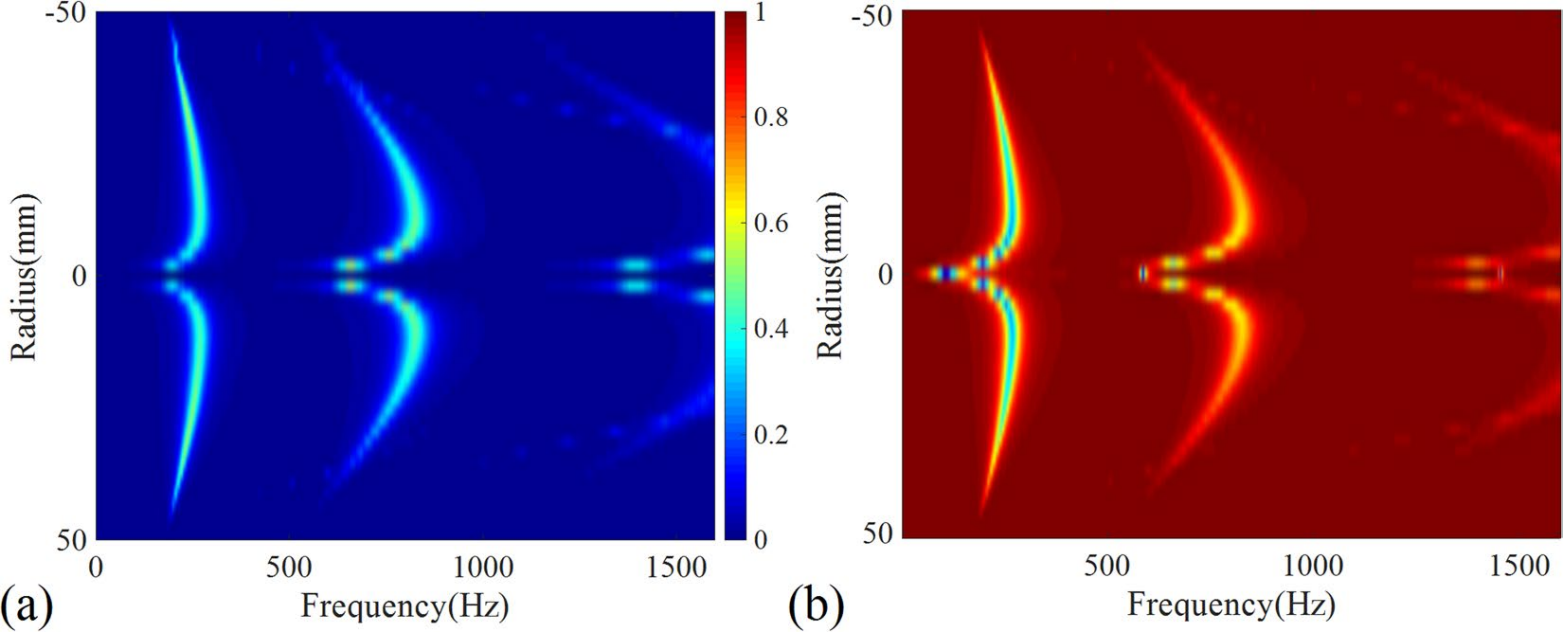
Parameter sweeping results. The sweeping color contours of the calculated sound (**a**) absorption coefficients and (**b**) reflection coefficients of the structures with the continuously changing in short-axis radius.

The above experiment and calculation results show that the STL, absorption and reflection coefficients of shell-type structures do not change monotonically with the curvature. To reveal the relationship between these acoustical parameters and the radius of curvature *R*, we calculated the distributions of the absorption and reflection coefficients as a function of frequency for structures with a continuously changing short-axis radius *r*(*r*∝1/*R*) through parameter sweeping. The results are shown in Fig. 4, in which it can be seen that the positions of the sound absorption peaks or the reflection dips of structures with a positive short-axis radius (concave side incidence) and of those with a negative short-axis radius (convex side incidence) are almost consistent. Using the sound absorption coefficient as an example, as the *r* increases, the frequency of the absorption peak first increases, then decreases, and this trend is wing shaped. In addition, the radian of the wing of the higher-order sound absorption peaks is greater than that of the lower-order peaks. The results also suggest that, in addition to the presence of three sound absorption peaks, there are some additional absorption peaks within a certain short-axis radius. For example, in Fig. 3a, there are multiple sound absorption peaks for A2A and A3A. In summary, the results of Fig. 4 clearly reveal the nonlinear wing shape in the relationship between absorption peaks and reflection dips with a short-axis radius. It’s worth noting that there is a blue dot when the radius is close to 0 at about 100 Hz in Fig. 4b, which means the reflection coefficient is almost 0. However, in the corresponding position of Fig. 4a, there is no red dot, it means this reflection dip is caused by a transmission peak, rather than an absorption peak. According to the vibration theory relating to the thin shell^42^, its resonant frequencies are proportional to *R/t*, where *R* and *t* are the curvature radius and the thickness of the shell, respectively. Thus, since the thickness remains as a constant, the resonant frequencies of the structure will decrease inversely with the increase of the short-axis radius, which can qualitatively explain the phenomenon that the positions of the sound absorption peaks (corresponding to the resonance frequencies) in Fig. 4 increase with the short-axis radius in a wing-shaped trend. This means that the difference in acoustic characteristics between plates and shells is caused by the different vibration properties of these two types of structures, which is originated from the different geometric symmetries between plates and shells.

## Bilayer Shell-type Structure for Perfect Sound Absorption

**Figure 5:**
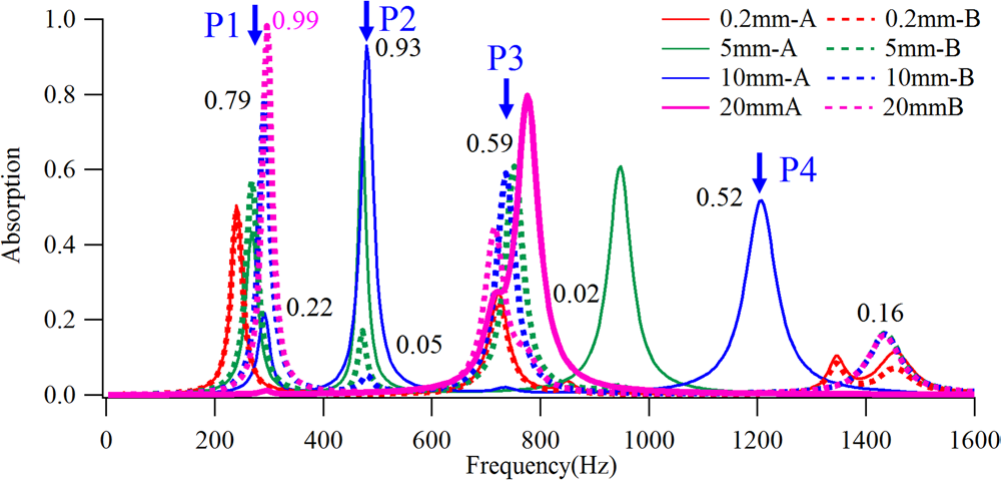
The calculated sound absorption coefficients of bilayer shell-type structures with different thickness of air gap and opposite incident directions.

As can be seen from Figs 3a, 4a, the sound absorption coefficients of all these structures are relatively low, with a maximum value of less than 0.5. Extensive previous research has determined that perfect sound absorption can be realized by the plate and membrane structures most similar to shells. Therefore, to fully extend the application range of shell-type structures^45^, in this section we hope to achieve perfect sound absorption through the shell-type structure. Previous work has shown that, by increasing the thickness of the air layer between the plate layers, a bilayer plate-type structure is able to achieve perfect sound absorption. For this purpose, we chose to use different four groups of distances, namely 0.2 mm, 5 mm, 10 mm and 20 mm, between the shell layers to design four groups of cylindrical shell-type structures. The absorptions under these different sound incidence directions were calculated and are plotted in Fig. 5. The figure shows that, with an increase of the distance between shells layers, on the one hand, the sound absorption coefficient at the sound absorption peak gradually increases, while, on the other hand, the bianisotropic feature becomes more obvious. When the distance was increased to 10 mm, the maximum sound absorption coefficient reached 0.93, and when it was continuously increased to 20 mm the largest sound absorption coefficient climbed to 0.99, thus demonstrating the perfect sound absorption effect. This, then, confirms that by properly selecting the distance between the shell layers, perfect sound absorption can be achieved by the bilayer shell-type structure.

In addition, the sound absorption coefficients in Fig. 5 indicate that, as the distance between the two shells increases, the bianisotropic sound absorption coefficient becomes increasingly stronger. Taking two sets of structures with a distance of 10 mm as an example, four sound absorbing peaks appear for each of 10 mm-A and 10 mm-B within the calculated frequency range. Among them, although the frequency positions of the first three peaks of the two sets are consistent, their absorption values are quite different. At point-P1, the absorption coefficient of 10 mm-A is only 0.22, while that of 10 mm-B is 0.79. At point-P2, the absorption coefficient of 10mm-A is as high as 0.93, while that of 10 mm-B is only 0.05. These results are equivalent to the former exhibiting a strong absorption, while the latter exhibits a strong reflection. At point-P3, the absorption of 10 mm-A is as low as 0.02, while that of 10 mm-B is higher and reaches to 0.59, demonstrating that, due to the asymmetry geometric feature, when the sound waves are incident from different sides, strong absorption and strong reflection effects can be respectively achieved. Moreover, the fourth absorption peak of 10 mm-A appears at point-P4, wherein the absorption value is 0.52, while that of 10 mm-B appears at a higher frequency, for which the absorption is only 0.16. This indicates that, as the direction of the incident wave changes, in addition to the different absorption magnitude, the frequency position of the absorption peak becomes different as well.

**Figure 6:**
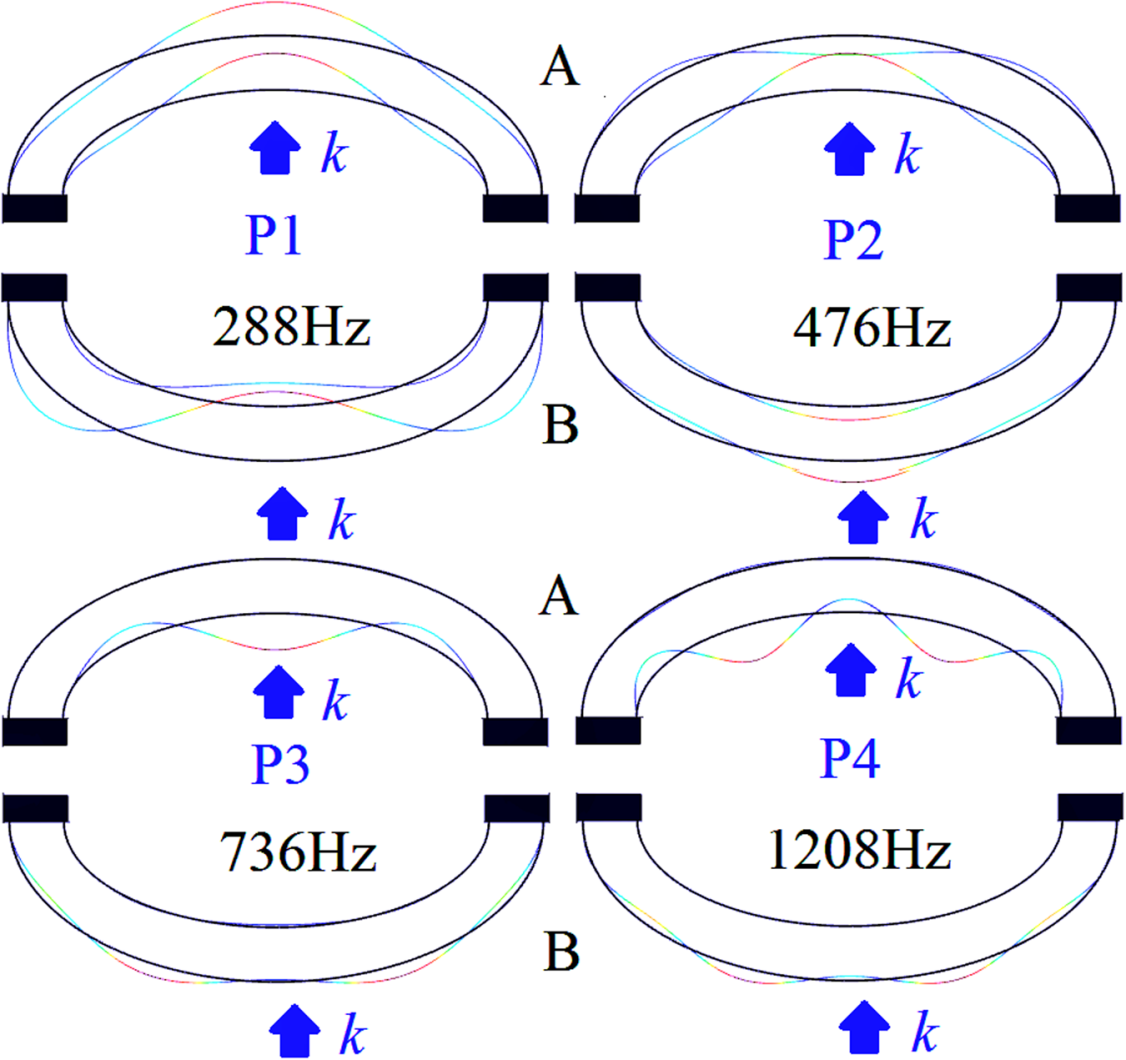
The displacement profiles at the points marked in Fig. 5 when sound wave incident from the concave side (up row, A) and the convex side (down row, B).

**Figure 7:**
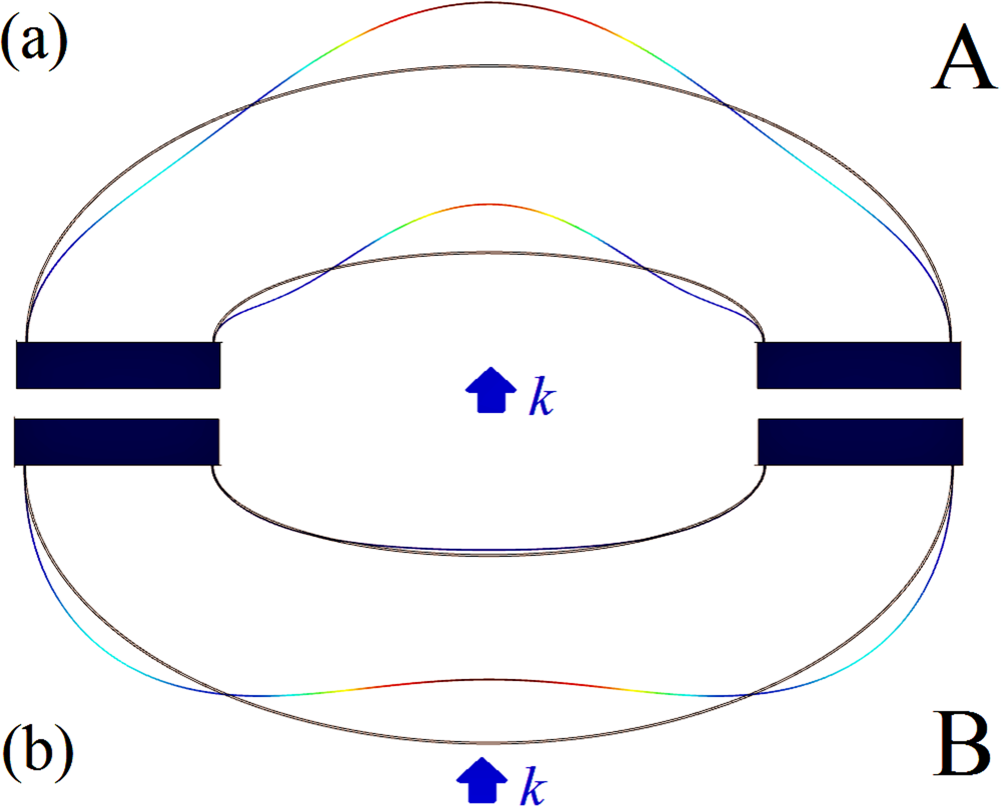
Displacement profiles at perfect absorption point. The displacement profiles at the point with perfect absorption when sound wave incident from the (**a**) concave side (A) and (**b**) convex side (B).

To clearly reveal this bianisotropic sound absorption mechanism, Fig. 6 shows the displacement profiles of the two sets of structures at the four points, from P1 to P4. From the figures it can be seen that, due to the asymmetrical geometrical characteristics of the shell-type structure, the displacement distributions under the opposite incident directions are totally different at all absorption peaks, making it equivalent to two different structures. In other words, due to its asymmetrical geometrical characteristics, the acoustic property of the shell-type structure is dependent on the sound incident direction and it exhibits a bianisotropic sound absorption and reflection characteristic. In addition, in order to reveal the mechanism of perfect sound absorption, the displacement profiles at point-P5 when the sound wave is incident from the convex side and the concave side were plotted, as shown in Fig. 7. In this it is evident that, when the sound wave is incident from the concave surface (group A), the displacement direction of the inner shell is the same as the incident sound wave. Moreover, the sound wave is easily transmitted, so the sound absorption coefficient is low. However, when the sound wave is incident from the convex side (group B), the displacement direction of the outer shell is the same as the incident acoustic wave, which produces resonance and is beneficial to impedance matching, but the inner shell remains almost stationary and the sound wave is difficult to transmit, and, therefore, a high sound absorption coefficient or even a perfect absorption can be obtained. Since the single structure can respectively achieve strong absorption and strong reflection under the opposite sound incident directions, these structures demonstrate potential for applications in the control of spatial sound field.

## Shell-type Skin Carpet Acoustic Cloak

Acoustic cloak is an important application topic for acoustic metamaterials. In previous work, we realized a planar carpet cloak through bilayer acoustic metasurface units^32^. At present, the proposed acoustic metasurface structures are planar, and can only realize a planar cloak, or approximately realize a cloak for an object with an arbitrary shape, through the approximation of an arc curve by segmented straight lines^41^. However, the surfaces of commonly used structures in practice are non-planar and, therefore, in this section, we hope to realize an arc-shaped carpet cloak that fits perfectly into a surface curve by means of a bilayer shell-type metasurface. The generalized Snell law is the basis of phase regulation of acoustic metasurfaces. Previous research suggests that the required phase shifts at different positions on the surface change with the different *h*, and the corresponding phase compensation of each cell could be solved by the following relation^6^:

3 $${\delta }_{i}=\pi -{\rm{2}}{k}_{0}{h}_{i}\,\cos \,\theta $$

in which *k*_0_ = 2π/*λ* is the wave vector in air, and *λ* is the manipulation wavelength.

**Figure 8:**
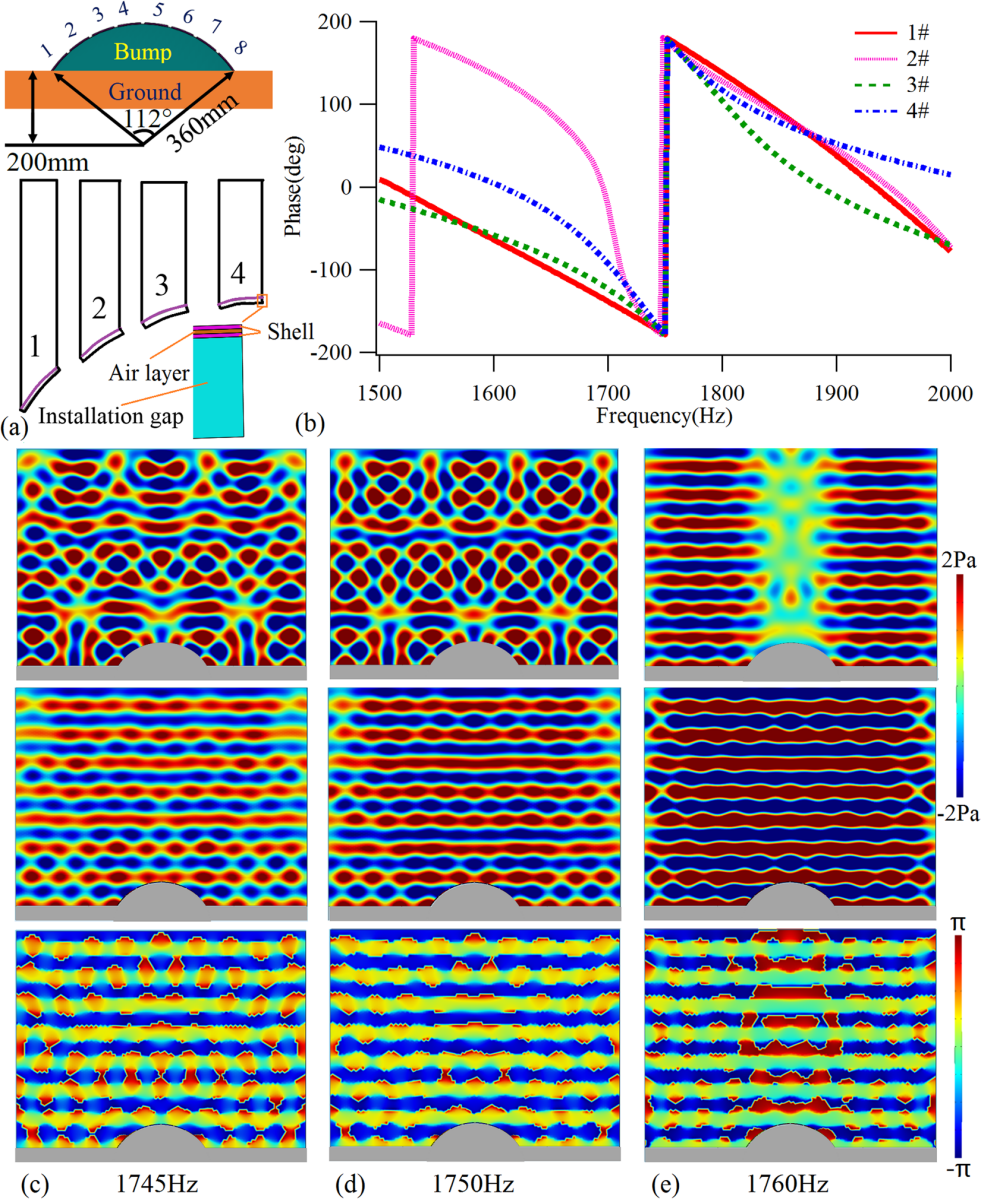
Arc-shape acoustic cloak. (**a**) Schematic of the arc-shape cloaked bump structure and its geometric parameters. (**b**) The reflection phases of the designed samples with iso-phase shifts at 1750 Hz. Pressure field distributions when an incoming sound plane wave perpendicularly illuminate the bare bump (up row), the arc-shape cloaked bump (middle row), and the phase contours for the case with a cloak (down row) at (**c**) 1745 Hz, (**d**) 1750 Hz, and (**e**) 1760 Hz.

Here, we select a sector structure as a hidden object. The radius of the structure is *R* = 360 mm, the distance from the bottom edge to the center of the circle is *H* = 200 mm, and the central angle of the sector is 112°, as shown in Fig. 8a. The cloak is constituted of 8 bilayer shell-type cells arranged symmetrically on the sector surface, in which the identical thickness of the rigid shells and air layer between shells is 0.2 mm, the installation gap is 4.4 mm, and the operating frequency is 1750 Hz. Due to the special characteristics of the shell-type structure, the detail structure between the cells is not consistent, and the phase compensation cannot be designed by the Eq. 3. Therefore, to realize the phase shift design, a new iso-phase design method is proposed to adjust the phase shifts for these complex surface structures. The main objective here is to establish the calculation models of different single cells in turn, according to the cell structure used in the actually cloak. Thereafter, the elastic modulus of the shells can be adjusted to realize the same phase shifts of all cells at operating frequency in the monitoring plane with same height to ground plane. The Young’s modulus of the plates is (5.94 + 0.297i) GPa for cells 1# and 8#, (6.22 + 0.311i) GPa for cells 2# and 7#, (3.88 + 0.199i) GPa for cells 3# and 6#, and (3.18 + 0.159i) GPa for cells 4# and 5# cells, respectively. Firstly, the phases of the single cells are solved, with those of the four cells on the left side shown in Fig. 8b. Moreover, a model with a bare arc-shape bump and a model with skin cloaked arc-shape bump are established based on the designed cell structures. The calculated pressure field distributions when an incoming sound plane wave perpendicularly illuminates the bare bump (up row) and cloaked bump (down row) are shown in Fig. 8c,e at 1745 Hz, 1750 Hz and 1760 Hz, respectively. These results suggest that the sound pressure distributions of a skin cloaked structure are close to the planar wave field within the narrow band of 1745~1760 Hz, which indicates that such a skin layer has obvious scattering reduction effect as a cloak. The corresponding mechanism for cloak performance can be intuitively revealed by the phase profiles, shown in Fig. 8b, which suggest that the phases of the four cells substantially coincide with each other in this frequency range, demonstrating that they have almost the same reflection phases as the monitoring height. Moreover, the scattering reduction effect as a cloak in Fig. 8c,e is not good enough, the main physical reason is that the gradient of height range covered by each unit is too large. If more cells with smaller sizes are selected, each cell will be closer to the planar plate structure, which is expected to improve the scattering reduction effect. It is worth noting that although the scattering reduction effect at 1760 Hz is better than the operating frequency of 1750 Hz in the pressure field distributions, it can be seen from the phase contours that the phase distortion at 1760 Hz is significantly higher than that at 1750 Hz. Therefore, in general, the cloaking effect at the operating frequency is better. Because this ultra-thin metasurface acoustic cloak can be flexibly arranged based on the surface of objects with complex surface topography, and the thickness is very small, the application ranges can be greatly extended.

## Conclusions

This paper proposes a shell-type acoustic metasurface that is expected to extend the current research results of membrane- and plate-type acoustic metamaterials to include shell-type structural systems, and to extend the application ranges of acoustic metamaterials in engineering practice. The sound insulation performance measurement and calculation results of the rotary spherical shell and the rotary ellipsoidal shell cell structures show that the sound insulation performances of the shell-type structure are quite different from those of the plate-type structure. When the curvature radius is reduced to a certain range, the cutoff frequency feature between the stiffness control region and the damping control region of the sound insulation curve can break, so the excellent broadband low-frequency and middle-frequency sound insulation performance could be obtained. Moreover, through the design of the widely used two-dimensional bilayer cylindrical and elliptical shell structures, the absorption and reflection coefficients, as well as the dynamic effective parameters of structures with different short-axis radius under different incident direction were calculated. The results indicate that, due to its asymmetrical geometric characteristics, the shell-type structure possesses bianisotropic acoustical parameters, and, moreover, that the stiffness of the shell-type structure changes nonlinearly as a wing shape with the radius of curvature. In addition, the calculation results show that the bilayer shell structure can achieve bianisotropic perfect absorption once the distance between the two shell layers has been properly increased to a certain value. Most importantly, the design of a bilayer cylindrical shell-type acoustic metasurface and use of a new engineered iso-phase design method, allows phase compensation for cell structures with different shapes to be adjusted to realize an arc-shape carpet cloak. Since such a shell structure can be designed according to the surface curve shape of the object, a carpet cloak that completely matches the object surface can be realized, and it is possible to design this carpet cloak with an arbitrary shape. However, compared with other types of cloak, the scattering still obvious in this arc-shape cloak, and there are some spaces to further improve the performance.

## Methods

### Experiment measurements

The experiment samples shown in Fig. 1b,c were fabricated by a photosensitive resin material through 3D printing. The STLs shown in Fig. 1d were measured by a Brüel & Kjær Type-4206T impedance tubes system.

### Numerical simulations

The simulations were performed with the commercial finite element analysis solver Comsol Multiphysics R4.3a. In the simulations in Figs 1, 2, two-dimensional axisymmetric module is employed to reduce the calculation cost, while in the simulations in Figs 3–8, two-dimensional module is used. For all calculations, plane wave radiation boundary conditions are set at the input and output planes of the air domains.
